# A Mediating Model of Emotional Balance and Procrastination on Academic Performance

**DOI:** 10.3389/fpsyg.2021.665196

**Published:** 2021-10-13

**Authors:** Pierluigi Diotaiuti, Giuseppe Valente, Stefania Mancone, Fernando Bellizzi

**Affiliations:** Department of Human Sciences, Society, and Health, University of Cassino and Southern Lazio, Cassino, Italy

**Keywords:** academic performance, procrastination, self-regulation, emotional balance, mediation model

## Abstract

Deficit in the management of time continues to be an important difficulty students are faced with. The present work aimed to test the hypothesis that self-regulation is the major predictor of academic performance and that this effect can be mediated both by the student’s emotional regulation and his propensity for procrastination. Participants were 450 university students who were administered MPP and AIP. The procedures involved the administration of instruments and the collection of average exam grades as a measure of academic performance. The effect of a specific component of self-regulation on academic performance, namely action orientation, was significant, while procrastination showed a limiting effect on the performative quality of the student. The model confirmed the mediation role of emotional balance on the effect that action orientation exerts on procrastination, and the mediation of procrastination in the relationship between action orientation and Academic Performance. Results of the study suggest focusing on student support and on prevention of procrastinating behavior through programs that enhance first of all student’s proactive attitude, planning skills, self-monitoring and effective/efficient time management, and secondly, emotional awareness and regulation of emotional response in situations of stress and performance anxiety.

## Introduction

Traditionally, procrastination was defined as any behavioral delay in starting or finishing a task (Solomon and Rothblum, 1984; Rothblum et al., 1986; Beck et al., 2000; Meyer, 2000; Ferrari et al., 2005; Klassen et al., 2008). Academic procrastination, according to Steel (2007a) can be defined as the deliberate delay in completing academic assignments even though one is aware of its negative outcomes and consequences (Steel, 2007b). The prevalence of chronic procrastination in students has been reported in numerous studies (Madhan et al., 2012; Day et al., 2014; Kim and Seo, 2015; Visser et al., 2018).

Researchers have consistently identified between 40% and 60% of students as involved in procrastination to a moderate or high degree (Onwuegbuzie, 2004). This high prevalence of procrastination appears to be observed similarly across cultures, as reported in the United States (Solomon and Rothblum, 1984; Rothblum et al., 1986; Onwuegbuzie, 2004), Canada and Singapore (Klassen et al., 2009), and Turkey (Özer et al., 2009), Iran (Mohammadi Bytamar et al., 2018). Lasane and Jones (2000) examined the relationship between temporal orientation (the predominant tendency to focus attention on a particular region of temporal space) and the frequency of self-reported academic procrastination. They hypothesized that students classified as either high in present time orientation or high in future time orientation would differ in their likelihood of experiencing socially induced temporal myopia, namely the degree to which short-term social events would interfere with academic goal-setting. High in present time oriented students resulted to procrastinate more and had worse performance. Often the student finds himself to starts working shortly before the deadline, procrastinating until the last possible moment to start an activity; when they have to prepare for an exam, they think they are taking less time than they have available, they concentrate their efforts towards the end of the estimated time and are often reduced to working in a hurry to make a last-minute submission (Patrzek et al., 2012).

Procrastination could even be considered a tactic to protect the subject’s vulnerable self-esteem. Various studies have shown a significant association between procrastination, low confidence and self-esteem, high levels of anxiety, depression, neurosis, forgetfulness, disorganization and lack of energy (Effert and Ferrari, 1989; Stöber and Joormann, 2001). Procrastinators are apparently more inclined to self-handicapping behavior; they use techniques that manipulate the impression they make on others and avoid any information that will lead to a realistic diagnosis of themselves (Ferrari, 1991).

Some researchers state that procrastination is highly correlated with the failure of self-regulation (Baumeister et al., 2007; Howell and Watson, 2007; Steel, 2007a; Pychyl and Flett, 2012; de Palo et al., 2017; Ziegler and Opdenakker, 2018). Baumeister and Heatherton (1996) have defined the failure of self-regulation as a wrong action committed by a person (sub-regulation) or an individual taking ineffective action (wrong self-regulation) in an attempt to initiate, alter or inhibit behavior. Some researchers approach procrastination as a form of sub-regulation: procrastinators fail to set standards, monitor performance and maintain motivation (Ferrari, 2001; Rabin et al., 2010). In the study by Garzón-Umerenkova et al. (2018) there is a description of the current models of the relationship between the variable SR (Self-Regulation) and procrastination (dysregulation). An interesting, recent general model that emphasizes the importance of the cyclical aspect of individual self-regulation (SR) and the external regulation derived from the context (ER), intended as different and complementary variables both in combination and interaction, is proposed by de la Fuente-Arias (2017) as Theory of Self- vs. Externally-Regulated Learning. As suggested by de la Fuente et al. (2020), Self-Regulation (SR) should be considered a personalistic variable or meta-ability and a behavioral materialization of personality factors. The study by de la Fuente et al. (2015) is significant with regard to the relationships between SR (considered a metabehavioral variable) and other meta cognitive and meta emotional variables. Other researchers believe that procrastinators engage in misregulation and procrastination actions as an emotional regulation strategy to repair bad moods caused by the task at hand (Tice et al., 2001; Sirois and Pychyl, 2013). Emotion regulation plays a critical role in understanding the self-regulatory failure of procrastination. Individuals postpone or avoid aversive tasks in order to gain short-term positive affect at the cost of long-term goals (Tice and Bratslavsky, 2000). With regard to the details of this process, Sirois and Pychyl (2013) have suggested considering counterfactual thinking as an explanation of emotional misregulation that may promote procrastination. As aversive affective states have been shown to cue procrastination by misregulation, it was hypothesized that the ability to adaptively cope with aversive affective states reduces the risk of procrastination (e.g., Blunt and Pychyl, 1998). Eckert et al. (2016) aimed to clarify the role of emotion regulation skills in order to reduce the tendency of procrastination.

Regarding the field of academic procrastination, Garzón-Umerenkova et al. (2018) indicate in their structural relationship model that self-regulation predicts procrastination in students directly and their level of academic performance indirectly. The study also reports that compared to the gender variable, women tend to procrastinate less and show a greater involvement (flourishing) in academic performance. A specific in-depth study in the literature (also quoted in the same article) on the differences between men and women regarding the intensity and type of procrastination is reported in Özer et al. (2009). A further recent contribution showed that both different personality traits and gender play a significant role in behaviour (Zhou, 2020). Duckworth et al. (2019) have also shown that low self-control causes procrastination. Students with high self-control are aware of their learning process and are more successful in balancing and speeding up their tasks. Currently, there are also several lines of research that analyse individual non-cognitive factors that increase the prediction of academic performance. Greater emotional regulation and a better process of adaptability are useful to cope with academic stress and achieve academic success (Saklofske et al., 2012). With regard to the (moderate) association between socio-emotional intelligence and academic performance see the recent meta-analytic study by Sánchez-Álvarez et al. (2020). As regards the relationship between procrastination and emotional regulation the results of the study by Mohammadi Bytamar et al. (2020) are also significant, as they show that procrastination is positively associated with problems in emotional regulation. This finding is in line with models that consider emotion regulation as self-regulation failure in procrastination (Pychyl and Sirois, 2016).

Literature models on the one hand therefore converge in stating that procrastination is essentially determined by a failure of self-regulation (Baumeister et al., 2007; Howell and Watson, 2007; Steel, 2007a; Pychyl and Flett, 2012; Ziegler and Opdenakker, 2018), on the other hand, there is simultaneously a line of studies that considers the role of emotion regulation on procrastination behaviour to be decisive (Ferrari, 2001; Tice et al., 2001; Pychyl and Sirois, 2016; Mohammadi Bytamar et al., 2020). Starting from this theoretical framework, in this study we firstly hypothesized that the student’s emotional balance could have a mediating function on the relationship between self-regulatory competence and procrastination behaviour; secondly, considering also the measure of academic performance (the grades of the exams taken), we hypothesized a serial mediation model where, the main relationship between self-regulation and academic performance were mediated by the student’s emotional balance (first mediator) and procrastination tendency (second mediator).

## Materials and Methods

### Participants

450 Italian university students were involved in the study: 237 (52.7%) males and 213 (47.3%) females, M age 22.25, SD age 2.87. Students voluntarily agreed to participate in the study after being informed of its objectives and they all supplied an adequate compilation of the instrument. Participants covered a substantially equal number of students attending science (56%) and humanities courses (44%). As a criterion for inclusion, enrolment in the third year of the course was considered, in order to include participants who were not beginners but who already have a well-structured approach and attitude towards university study and were not conditioned by the transition from high school to university. The administration of the instruments was done through an on-site group procedure.

### Tools

Two tools were Administered:

1. *Multidimensional personality profile* (MPP; Caprara et al., 2006), developed from the two dominant models in the psychology of personality: the social-cognitive theory and the theory of traits. The test measures five fundamental areas of personality: Agentivity, Social–Emotional Intelligence, Self-regulation, Ability to Cope (with critical situations), Innovation. Each of these areas is divided into sub-dimensions that analyze its content by anchoring it directly to behaviors, subjective states and significant external criteria. The test consists of 152 statements with which it is necessary to express one’s degree of agreement on a five-level scale. For the purposes of this work, the Socio-emotional intelligence (Empathy, Prosociality, Sociability, Interpersonal Confidence), Self-regulation (Action Orientation, Tenacity, Reliability, Accuracy) and Ability to Cope (Emotional Balance, Stress Management, Negative Emotion Management, Resilience) scales were administered. Reliability measures through McDonald’s omega and Cronbach’s alpha were both 0.84, 0.82, 0.76, respectively. Considering the theoretical models of reference of the study and the mediation relations hypothesised, the choice of this instrument, compared to other personality tests validated in Italian, was motivated by the possibility of measuring in the student both self-regulation with its components (Action Orientation, Tenacity, Reliability, Accuracy), and the various aspects linked to the ability to cope and manage emotions.
2. *Adult inventory for procrastination* (AIP, McCown and Johnson, 1989; It.valid. Mariani and Ferrari, 2012); the AIP scale measures the chronic tendency to postpone tasks in various situations. It examines procrastination motivated by fears (e.g., success or failure), the avoidance of disclosure of skill inabilities, and performance insecurity. The tool assesses avoidance procrastination; that is, putting off tasks to protect one’s self-esteem from possible failure. The AIP is composed of 15 Likert-scale items such that respondents express an opinion on a 5-point scale (1=strongly disagree; 5=strongly agree) to statements such as “I am not very good at meeting deadlines” and “I do not get things done on time.” Both reliability measures for McDonald’s omega and Cronbach’s alpha were 0.80.
3. In addition to the administration of the psychometric tools, participants were asked to report their own academic performance, which corresponds to the average of the grades obtained in the examinations. University exam grades in Italy range from a minimum of 18/30 to a maximum grade of 30/30. In order to avoid being inaccurate in remembering their grade point average, students were asked to consult their digital profile on the university portal, where this data is available.

### Procedures

Students voluntarily agreed to participate in the study after being informed of its objectives and they all supplied an adequate compilation of the instrument. They were also informed of the anonymity of the test and the fact that it was designed for research purposes only. The protocol was approved by the *Institutional Review Board* of the local university. Tools administration took place upon the release and signing of the form for an informed consent of participation.

## Statistical Analysis

Descriptive statistics were performed to illustrate socio-demographic information (gender, age, study area); Pearson and Spearman bivariate correlations, significant at *p*<0.005 and at *p*<0.001 2-tailed, were used to measure the association between the main variables (Socio-Emotional Intelligence, Self-Regulation, Ability to Cope, Procrastination); Cronbach’s alpha and McDonald’s omega were considered as scale reliability coefficients; *T*-test was used to explore significance in procrastination behaviors relating to gender; ANOVA univariate test with *Post-hoc* Tukey HSD and *p*<0.05 to explore associations between Procrastination, Emotional Balance and Self-Regulation; a hierarchical regression was performed to identify the predictors of procrastination and academic performance; a simple mediation analysis was run to test the function of Emotional Balance on the effects of Procrastination on Academic Performance; a serial mediation analysis was run to test the effects of Emotional Balance and Procrastination in the relationship between Action-Orientation and Academic Performance. Association and prediction analyses were performed using the packages SPSS v. 22, while mediation analyses were performed through the PROCESS macro version 2.3^1^ (Hayes, 2017). As the Process macro procedure shows the unstandardized beta values, all varibles were standardized before to run mediational analyses.

## Results

### Relations of Association

The Table 1 shows the bivariate correlations between the main variables of the study.

**Table 1:** Variables means and bivariate correlations.

	Mean	SD	Sk	Ku	PR	SR	EB	AP
Procrastination	2.53	0.69	−0.24	−0.38	1			
Self-Regulation	3.38	0.48	0.40	−0.19	−0.455^**^	1		
Emotional Balance	3.18	0.73	0.26	−0.18	−0.400^**^	0.378^**^	1	
Academic Performance	24.81	2.31	0.21	−0.39	−0.287^**^	0.222^*^	0.191^*^	1
Socio-Emotional Intelligence	3.25	0.37	0.14	−0.11	−0.125	0.255^**^	0.286^**^	−0.253^**^

*SD, standard deviation; Sk*, *skewness; Ku*, *kurtosis.*

** *Correlation is significant at the 0.01 level (2-tailed).*

* *Correlation is significant at the 0.05 level (2-tailed). For AP Spearman’s correlation has been used. PR*, *Procrastination; SR, Self-Regulation; EB, Emotional Balance; AP*, *Academic Performance; EI*, *Socio-Emotional Intelligence. N=450*.

Taking into consideration performance in the studies, that is, the average grades of successful exams (whereas in Italy the academic grades range from a minimum of 18 to a maximum of 30), significant association resulted with the level of procrastination. A greater level of *Procrastination* was significantly associated with a lower *Academic Performance*: *F*(1,449)=3.981 *p*=0.04; *η^2^*=0.03.

Considering the level of procrastination within the sample as a function of gender, a significant result emerged upon examination of the *t*-student test: t(448)=2.457 *p*=0.02; *d*=0.40. Male procrastination was significantly higher (*M_m_*=2.66; SD=0.61) than the female one (*M_f_*=2.39; SD=0.74). It was then investigated whether the trait components helped to explain the overall procrastination orientation, also according to the gender of the participants. Among the MPP measures considered, significance was found in association with *Emotional Balance* and *Self-Regulation*. A greater level of Emotional Balance was significantly associated with a lower propensity for Procrastination: *F*(1,449)=37.937 *p*=0.000, *η^2^*=0.20; OP=0.99. At the same time, a greater level of Self-Regulation was significantly associated with a lower propensity for Procrastination: F(1,449)=21.020 *p*=0.000, *η*^2^=0.12; OP=0.99. The interaction between the two variables (Emotional Balance^*^Self-Regulation) was also significant: *F*(3,449)=8.948 *p*=0.003, *η^2^*=0.06; OP=0.84. Corrected model: *F*(3,449)=20.645 *p*=0.000, *η^2^*=0.30; OP=0.99.

Taking into account the males alone, among the MPP measures significance was found in association with *Emotional Balance*. A greater level of Emotional Balance was significantly associated with a lower propensity for Procrastination: *F*(1,236)=21.109 *p*=0.000; *η^2^*=0.21 OP=0.99. Whereas for the females, significance resulted between *Procrastination* and Self-Regulation. A lower level of Self-Regulation corresponded to a greater propensity for procrastination: *F*(1,212)=15.601 *p*=0.000; *η^2^*=0.18 OP=0.97.

### Predictors and Mediators of Procrastination

#### Relations of Prediction

Subsequently, in order to identify the predictors of Procrastination, a hierarchical regression was carried out by inserting as potential predictors the variables that had shown significance in the analysis of previous variance. The assumptions of multivariate normality were first verified: standardized mean residuals=0.000; Durbin-Watson=2.169; VIF<1.2; Tolerance >0.84. F(1,449)=22.192; *p*=0.000; R^2^=0.331. Significant predictors of Procrastination were a lower level of *Self-Regulation* (ΔR^2^=0.21; *β*=− 0.328), lower *Emotional Balance* (ΔR^2^=0.06; *β*=−0.303), and Gender (ΔR^2^=0.05; *β*=−0.217).

#### Mediation Relations

Considering the first hypothesis of the study, in order to assess the estimates of the relationship between predictors, a mediation analysis was carried out to test if emotional balance plays a mediating role in the relationship between self-regulation and student procrastination.

**Figure 1:**
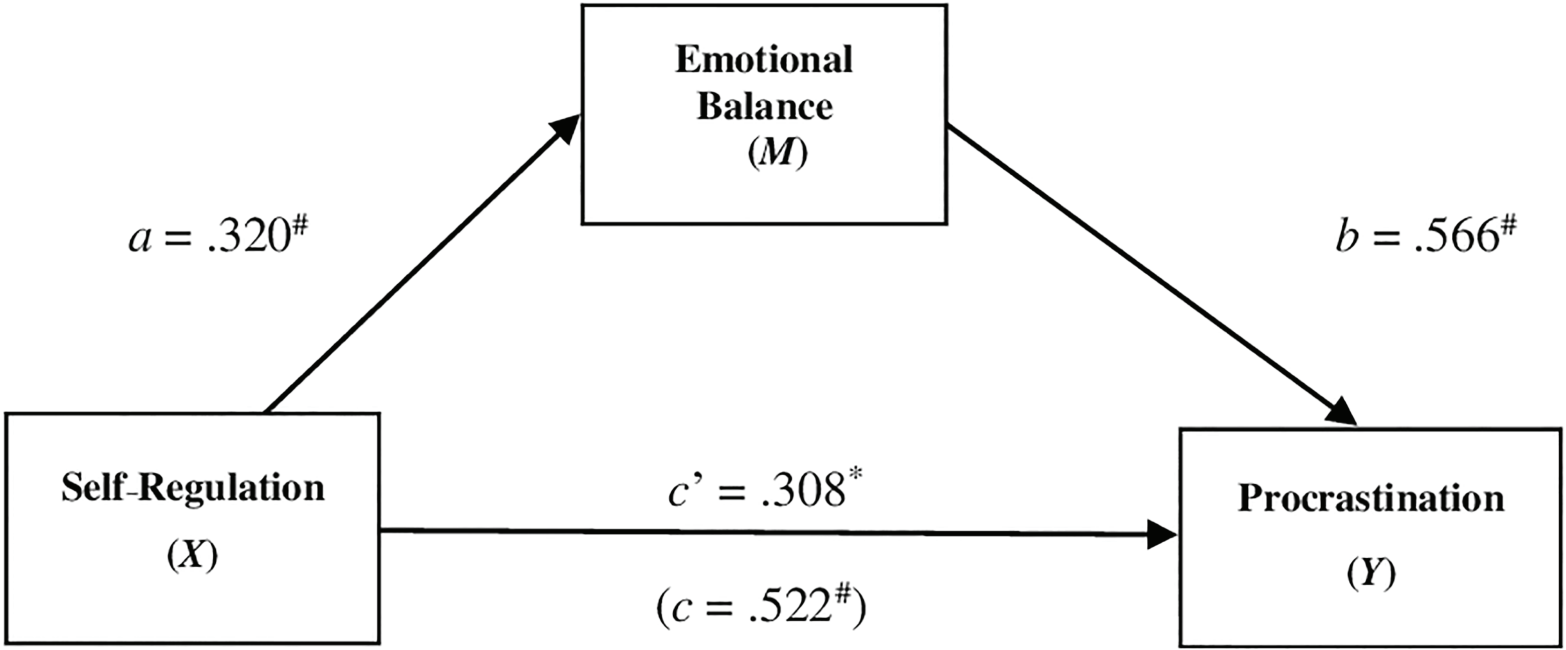
The mediating effect of emotional balance in the relationship between Self-Regulation and procrastination. ^#^*p*<0.001, ^*^p<0.05; all presented effects are unstandardized; *a* is effect of Self-Regulation on Emotional Balance; *b* is effect of Emotional Balance on Procrastination; *C*ʹ is direct effect of Self-Regulation on Procrastination. *C* is total effect of Self-Regulation on Procrastination.

Results from a simple mediation analysis indicated that Self-Regulation is indirectly related to Procrastination through its relationship with Emotional Balance. First, as can be seen in Figure 1, self-regulation has a positive estimate on the emotional balance (*a*=0.378, *p*=0.000), and a higher reported emotional balance was subsequently related to less procrastination (*b*=− 0.266, *p*=0.000). A 95% bias-corrected confidence interval based on 10,000 bootstrap samples indicated that the indirect effect (ab=− 0.100) was entirely below zero (−0.172 to −0.037). Moreover, higher levels of self-regulation corresponded to lower procrastination even after taking into account SR’s indirect effect through Emotional Balance (*C*ʹ=−0.513, *p*=0.000).

This result therefore confirmed the first hypothesis of the study.

### Predictors and Mediators of Academic Performance

#### Relations of Prediction

In order to identify the predictors of Academic Performance, a hierarchical regression was carried out by inserting as potential predictors the variables that had shown significance in the analysis of previous variance. Significant predictors of Academic Performance were a higher level of *Action-Orientation* (ΔR^2^=0.028; *β*=0.174), lower *Procrastination* (ΔR^2^=0.053; *β*=−0.179).

#### Mediation Relations

**Figure 2:**
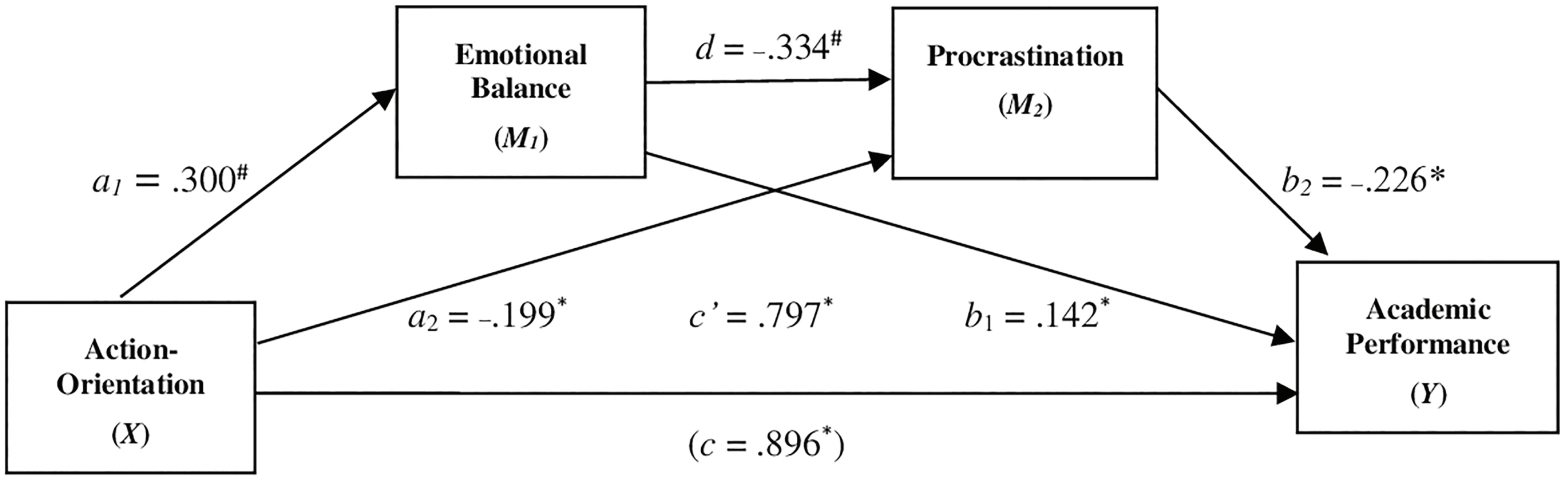
The serial mediating effect of *Emotional Balance* and *Procrastination* in the relationship between *Action- Oriantation* and *Academic Performance*. ^*^p<0.05, ^**^p<0.01, ^#^*p*<0.001; all presented effects are unstandardized; *a_n_* is effect of Action- Oriantation on mediators; *b_n_* is effect of mediators on Academic Performance: *c*ʹ is direct efffect of Action- Oriantation on Academic Performance; *c* is total effect of Action- Oriantation on Academic Performance; d is effect of emorional balance on procrastination.

Considering the second hypothesis of the study, a general explanatory model was tested through a serial mediation analysis of the effect of Action Orientation on Academic Performance. The results, as shown in Figure 2, indicated that Action-Orientation was indirectly related to Academic Performance through its relationship with Emotional Balance and Procrastination. First, as can be seen in Figure 2, Action-Orientation had a positive estimate on Emotional Balance (*a_1_*=0.300, *p*=0.000), and a higher reported Emotional Balance was subsequently related to more Academic Performance (*b_1_*=0.142, *p*=0.048). A 95% bias-corrected confidence interval based on 10,000 bootstrap samples indicated that the indirect effect through Emotional Balance (a_1_b_1_=0.043), keeping the other mediator constant, was entirely above zero (0.024 to 0.119). Second, Action-Orientation had a negative effect on Procrastination (*a_2_*=− 0.199, *p*=0.018), and a higher reported Procrastination was subsequently related to less Academic Performance (*b_2_*=− 0.226, *p*=0.016). A 95% bias-corrected confidence interval based on 10,000 bootstrap samples indicated that the indirect effect through Procrastination (a_2_b_2_=0.045), keeping the other mediator constant, was entirely below zero (0.003 to 0.103). Third, Emotional Balance (first mediator) had a negative effect on Procrastination (second mediator; *d*=−0.334, *p*=0.000), and the indirect effect through both mediators (a_1_db_2_=0.023) was entirely below zero (0.002 to 0.057). Furthermore, higher levels of Action-Orientation corresponded to higher Academic Performance even after taking into account Action-Orientation’s indirect effect through Emotional Balance and Procrastination (*C*ʹ=0.797, *p*=0.025).

This result therefore also confirmed the second hypothesis of the study.

## Discussion

The results of the study firstly confirmed the evidence in literature of the prevalence of procrastination behaviour in male students (Steel, 2007a; Özer et al., 2009; Steel and Ferrari, 2013; Mandap, 2016; Balkis and Duru, 2017; Limone et al., 2020). It was also found that in male procrastination there were reasons mainly related to emotional regulation, whereas in female procrastination there were significant self-regulatory aspects, such as those related to planning and time management, namely the action orientation. Probably in males it would affect a lower ability to use strategies to control states of anxiety and stress, sense of loneliness and frustration, depressive states and negative mood that often involve students in the college path. Goubet and Chrysikou’s (2019) recent study generally highlights a smaller repertoire of emotional regulation strategies and significantly less flexibility of implementation in males compared to females. Failing to fully accept and cope with their negative states, male students are likely to defend themselves through systematic avoidance, impulsively channeling their energies into social, sporting, playful, virtual, video games, and gambling activities, thereby distancing themselves from the academic task (Zhong, 2013; Petruccelli et al., 2014; Nordby et al., 2019; Shi et al., 2021). This impulsive tendency towards self-distraction and defocusing progressively lowers the level of engagement and motivation to study (Wypych et al., 2018; Svartdal et al., 2020). Instead, the results regarding the variables associated with female procrastination emphasize a limitation in the ability of self-management and pragmatic orientation towards the goals to be achieved. According to several studies, fear of failure and lack of self-regulated learning strategies would be the factors significantly attributable to female procrastination in university contexts (Özer et al., 2009; Abdi Zarrin et al., 2020; Limone et al., 2020). The tendency to perfectionism with its maladaptive aspects (see Ghosh and Roy, 2017; Newman et al., 2019), could also negatively affect a balanced and pragmatic assessment of the most effective actions to take towards the main goal. Action orientation is actually defined as the ability to plan and monitor a course of action in order to achieve a specific objective. Depending on the task and context in which it is found, the student will have to make an estimate of the time and effort required to carry out the assignment (see Sharaf et al., 2016; Alsalem et al., 2017). Nevertheless, deficit in time management continues to be an important difficulty that students have to face. Students with low results can make mistakes in evaluating how much study time is needed to assimilate the more difficult subjects (Broadbent, 2017; He and Zhang, 2019). Sometimes this is linked to the fact that students establish unrealistic objectives and are not in a condition to be able to evaluate the actual time needed for acquiring the skills and knowledge (Morgan, 1985; Gustavson and Miyake, 2017; Jalagat, 2017).

Results of the study showed also the significance of an initial model of mediation in which the student’s emotional balance played a role in mediating the effect of self-regulation on procrastination. Emotional balance represents the ability to control the influence of one’s mood and emotions on one’s behavior. This highlights that both the cognitive regulation component (SR) and the emotional regulation component are elements that could condition the procrastination behaviour, but above all that the student’s emotional balance can increase the effect of the control of organizing and planning the work that must be done to pass the exams.

Various studies have shown a significant association between procrastination, low confidence and self-esteem, high levels of anxiety, depression, neurosis, forgetfulness, disorganization and lack of energy (Effert and Ferrari, 1989; Garzón-Umerenkova et al., 2020). Procrastinators are apparently more inclined to self-handcapping behaviours; they use techniques that manipulate the impression they make on others and avoid any information that will lead to a realistic diagnosis of themselves (Ferrari, 1991; Ferrari and Tice, 2000). In addition, the relationship established with self-handicap behaviors, seems to provide evidence regarding the consideration of procrastination and self-handicap as a dysregulatory behavior, considered by the recent SRL vs. ERL Theory (de la Fuente-Arias, 2017).

The results of our study showed that emotional balance is a key competence for the student. They agree with Pychyl and Sirois (2016)and Wypych et al. (2018), who emphasize the role of emotion regulation and stress coping in procrastination. Procrastination was postulated to result from negative emotions, such as fear of failure (Schouwenburg, 1992) or discomfort intolerance (Harrington, 2005). Accordingly, highly procrastinating students were found to be much more sensitive to punishment than their non-procrastinating colleagues (Michałowski et al., 2017). Additionally, various negative emotions related to tasks were shown to lead to the avoidance of those tasks (Blunt and Pychyl, 2000).

The second model in our study included a performance measure relating to the average marks that students received at the examinations. As can be seen in Figure 2, with regard to the performance variable the estimate of a specific component of self-regulation, namely action orientation, was significant, while procrastination reported a negative association with the performative quality of the student. This result is consistent with other recent studies (Hen and Goroshit, 2014; Kyung and Eun, 2015; Yilmaz, 2017; Asadzadeh, 2018; Goroshit, 2018; Bashir and Gupta, 2019). The model reported a mediation role of emotional balance on the estimated effect of action orientation on procrastination; at the same time, a negative and significant relationship between emotional balance and academic performance. This suggests that a low regulation capacity of the emotional sphere would indirectly have (*via* procrastination) consequences of limiting the quality of academic performance. These relationships would distinguish this study from those which have instead only hypothesized a direct relationship between emotional dysregulation and academic performance (see Crego et al., 2016; Frazier et al., 2018; Vizoso Gómez et al., 2018; Morales-Rodríguez and Pérez-Mármol, 2019).

As already pointed out by Dresel et al. (2015), resources management strategies, i.e., the ability to manage external resources, are just as important as internal emotional control., as in seeking for help, or organizing one’s workplace, as well as to managing and regulating internal resources, such as effort regulation, time management, attentional regulation, and motivation. In the university context of the last two years, the transition from face-to-face to remote teaching, as a result of the limitations of the covid-19 pandemic, has strongly emphasized the need for students to possess pronounced self-management skills in their learning processes in order not to fall behind on scheduled paths and deadlines and also to maintain a good level of perceived psychological well-being (Pelikan et al., 2021; Diotaiuti et al., 2021a). It has been observed in several studies how distractions at home and reduced social interaction with other students and faculty can negatively affect study time management and adaptation to the new emergency condition that requires students to have a higher level of autonomy and metacognitive control (Rasheed et al., 2020; Tezer, 2020; Alyami et al., 2021; Biwer et al., 2021; Diotaiuti et al., 2021b).

## Implications of the Study

The results of this study suggest that student support and procrastination behavior prevention interventions should focus on programs that enhance first of all the student’s proactive attitude, planning skills, self-monitoring and effective/efficient time management, and secondly, emotional awareness and regulation of emotional response in situations of stress and performance anxiety.

In the literature, as reported by Goroshit (2018), few programs have focused on limiting student procrastination (Bui, 2007; Rakes and Dunn, 2010; Grunschel et al., 2016; Stewart et al., 2016; Deleval et al., 2017); but emotion regulation training has been shown to reduce procrastination (see Eckert et al., 2016; Van Eerde and Klingsieck, 2018). Awareness of the problem should therefore be accompanied by a willingness to build and validate effective programs in the field. The literature has indicated that it is also necessary to consider potentially influential situational variables, such as the degree of satisfaction associated with the learning context, living (or not) in a university residence, a distance learning environment, the specific needs of the person, individual beliefs and expectations (Rustemeyer and Rausch, 2007; Medrano et al., 2016; Yilmaz, 2017; Goroshit, 2018). An innovative and effective intervention program should therefore include the control and manipulation of these aspects.

Students should be guided by experienced counsellors to increase their effort and persistence, and optimize self-control together with emotional balance. In this regard, it is important to use a communicative style adapted to the current needs of students and able to functionally affect their attitudes towards study and their life project (Daulay et al., 2018; Maddineshat et al., 2019; Diotaiuti et al., 2020). Such target programs could improve not only students’ academic life, but also their general well-being. The work on students’ self-regulation concerns the enhancement of the individual’s capacity for planning and persistence in achieving a goal, self-discipline, understood both as a self-reflective ability to organize and as tenacity for success, a love of order and precision which is distinguished by the desire to do things well (Colthorpe et al., 2017; Broadbent and Fuller-Tyszkiewicz, 2018; Bernardo et al., 2019).

Future studies could also try to understand if and how students’ temporal perspectives or temporal foci may also play a role among the predictors of university procrastination (Baguri et al., 2020; Levasseur et al., 2020; Diotaiuti et al., 2021c). In this case, a university couselling programme should also include an in-depth examination of the value of the person’s temporal perspective and the importance of the focus of attention that the student mainly devotes to the declinations of his/her own time (past, present and future).

## Limitations of the Study

Although this study yielded significant findings, some limitations should be considered. First, the use of self-report measures, exposed to the risk of underestimation of the reported procrastination reported by the students. Moreover, this was a cross-sectional research study in which the data were collected in a specific time period. Therefore, it is suggested that further research be carried out longitudinally to determine the level of procrastination behaviors among students, by also expanding the sample to students from other universities and geographical areas. Given the cross-sectional design, the research dealt with the associations between self-regulation, emotional balance, academic procrastination, and academic performance, and not with causes and effects. Nevertheless, given that the cross-sectional design does not inform on causation, the obtained mediations are an estimate that does not contradict the mediations relationships, although they do not prove them (Maxwell and Cole, 2011). Results should therefore be interpreted considering that the effects are compatible with the hypothesized theoretical models, while the direction remains open and this must be possibly demonstrated both theoretically and with more suitable research designs. Future investigations should carry out experimental designs to examine more specifically effect directions.

Finally, a further limitation is that this research is located at a molecular level and therefore does not allow the integration of real contextual variables of the teaching process, which are very important in the psycho-educational analysis of self-regulation and procrastination. Consequently, a further level of analysis is needed in order to offer a contextualized, interactive view of the object of study.

## Data Availability Statement

The raw data supporting the conclusions of this article will be made available by the authors, without undue reservation.

## Ethics Statement

The studies involving human participants were reviewed and approved by Institutional Rewiew Board of the University of Cassino and Southern Lazio. The patients/participants provided their written informed consent to participate in this study.

## Author Contributions

PD, GV, and SM designed the study. PD, GV, and SM analyzed the data and discussed the results. PD, GV, and FB drafted the manuscript. SM, FB, and GV revised the manuscript. Finally, the authors have agreed to be accountable for all aspects of the manuscript in ensuring that questions related to the accuracy or integrity of any part of it are appropriately investigated and resolved. All authors read and approved the final manuscript.

## Conflict of Interest

The authors declare that the research was conducted in the absence of any commercial or financial relationships that could be construed as a potential conflict of interest.

## Publisher’s Note

All claims expressed in this article are solely those of the authors and do not necessarily represent those of their affiliated organizations, or those of the publisher, the editors and the reviewers. Any product that may be evaluated in this article, or claim that may be made by its manufacturer, is not guaranteed or endorsed by the publisher.

## Footnotes

^1^ www.processmacro.org
